# Nursing Homes: Criticism with Suggestions

**Published:** 1919-02-15

**Authors:** Gladys M. E. Leigh


### Nursing Homes: Criticism with Suggestions.

*To the Editor of* The Hospital.

Sir,—Now, when there is so much demand for reconstruction in the nursing world, surely the time has come for revision in the arrangements of private nursing homes. Since demobilisation I have been working in a West-End home. I am struck by two facts: (1) The discontent of the personnel; (2) The lack of organisation. The owners, anxious to increase their takings, admit more patients than they can comfortably accommodate. The extra work is thrown on the nursing staff, who receive no increased emolument and find their off-duty curtailed. The domestic arrangements are in the hands of the owners' relatives, obsessed with the same idea and guiltless of method. The first difficulty could be overcome by granting the nursing staff a bonus on the profits. The second, by appointing a sister-in-charge of the kitchen arrangements certificated in the science of domestic economy.

Yours faithfully,

Gladys M. E. Leigh.

[Nurse Leigh's terse statement affords much information of an illuminating kind to those who have made it their business to study the abuses attaching to nursing homes as they exist to-day. The latter realise that with the advent of a Ministry of Health the day of nursing homes may shortly be ended, unless the county councils will make it their business to take powers for the periodical inspection of every such home by competent officers, with full authority to close where found necessary, and in any case to protect the public from overcharges, and to insist upon the patients being made the first consideration in any homes which are allowed to continue, as they are in this country in all well-administered voluntary hospitals. There is a class of so-called nursing homes that have been and are a grave scandal. Some of them sweat their nurses, over-charge the patients, and are, there is good reason to believe, places which should be promptly and finally put an end to. It would be very interesting and instructive if the municipalities and county councils throughout Great Britain would make it their business to secure and to have published without delay the history, growth, and present internal condition of every nursing home throughout the country, the number of women dressed in nursing costume who are not trained certificated nurses, the hours of the working staff, whether trained or otherwise, the payments made to every class of worker employed at these homes, the life-story of the owners and managers of such places, the amount of money taken and the profits made during each of the last three years in every case. Nurse Leigh has only touched the fringe of the subject. It should be the privilege of the Ministry of Health to tear aside all camouflage and deception, and, in co-operation with the municipalities and county councils, speedily to remove a multitude of existing evils in this connection which have too long been suffered and ought never to have been permitted to exist.—Ed. The Hospital.]

